# Meteorology

**Published:** 1836

**Authors:** 


### Art. II. — Meteorology.

1. *Abstract of Meteorological Observations, for the Calendar year* 1835. By Professor Ray, of Woodward College, Cincinnati.

### Editorial Remarks.

The spot where the observations, (the results of which are stated in the following tables,) were made, is on the plain north of the Ohio river, nearly a mile from the margin of the stream; about fifty feet above high water mark; and five-hundred feet to the south-west of the range of hills, which rise nearly three hundred feet above the plain. Its elevation above the level of the sea, is not far from five hundred and forty feet. Lat. 36 deg. 6 min. N.—Long. 84 deg. 27 min. W. The contiguity of this place of observation, to the hills which lie immediately to its north-east, would seem calculated—in consequence of their reflection in summer, and radiation in winter—to keep the temperature of both, a little higher than it would otherwise be; but a comparison of the results obtained by professor Ray with others, does not show that kind of influence; for in five continuous years, that the editor of this Journal, formerly, made meteorological observations, in another part of the city, the mean annual heat never fell below 52°.65, while that of 1835, as may be seen in the table, was 51°.3. That year was, indeed, taking it altogether, a cold year, as professor Ray has remarked; and brought forth one day, February 8th, that had not been equaled, in Cincinnati for thirty-eight years;—that is, since the 8th of January, 1797, when according to Governor Sargent, the mercury fell to 18 deg. below zero, *one degree lower* than it fell last year.

ABSTRACT.

| | Thermometer | | | | Weather | | | Rain & melted snow | | Barometer | | | |
|---|---|---|---|---|---|---|---|---|---|---|---|---|---|
| | Maximum | Minimum | Range | Mean Temp. | Clear & fair days | Variable | Cloudy | Inches | 100ths | Maximum | Minimum | Range | Mean |
| January. | 66 | 3 | 63 | 34.6 | 13 | 8 | 10 | 3 | 82 | 29.74 | 28.73 | 1.01 | 29.310 |
| February. | 56 | -17 | 73 | 24.5 | 6 | 12 | 10 | 1 | 75 | 29.79 | 29.05 | .74 | 29.460 |
| March. | 70 | 1 | 69 | 40.1 | 16 | 9 | 6 | 1 | 86 | 29.89 | 28.74 | 1.15 | 29.368 |
| April. | 83 | 21 | 62 | 50.5 | 9 | 11 | 10 | 3 | 37 | 29.58 | 28.91 | .67 | 29.249 |
| May. | 91 | 40 | 51 | 65.3 | 11 | 13 | 7 | 7 | 57 | 29.51 | 28.94 | .57 | 29.286 |
| June. | 95 | 45 | 50 | 71.2 | 8 | 16 | 6 | 7 | 34 | 29.51 | 29.04 | .47 | 29.304 |
| July. | 93 | 48 | 45 | 71.7 | 11 | 16 | 4 | 2 | 46 | 29.53 | 29.18 | .35 | 29.346 |
| August. | 89 | 46 | 43 | 69.1 | 9 | 16 | 6 | 6 | 54 | 29.52 | 29.13 | .39 | 29.338 |
| September. | 86 | 33 | 53 | 59.1 | 10 | 12 | 8 | 3 | 23 | 29.66 | 28.79 | .87 | 29.371 |
| October. | 82 | 29 | 53 | 55.8 | 16 | 8 | 7 | 4 | 35 | 29.73 | 28.96 | .77 | 29.403 |
| November. | 76 | 3 | 73 | 43.3 | 6 | 9 | 15 | 6 | 66 | 29.67 | 28.70 | .97 | 29.334 |
| December. | 63 | 9 | 54 | 31.4 | 12 | 5 | 14 | 2 | 20 | 29.75 | 28.92 | .83 | 29.464 |
| | | | | 51.3 | 127 | 135 | 103 | 52 | 15 | | | | 29.3528 |

WINDS.

| | N. | N. E. | E. | S. E. | S. | S. W. | W. | N. W. |
|---|---|---|---|---|---|---|---|---|
| January. | 2½ | 9½ | 2 | 3½ | 1½ | 5 | 4 | 3 |
| February. | 0 | 3 | 0 | 1 | 1 | 7 | 9½ | 6½ |
| March. | 1½ | 3½ | 2½ | 1½ | 2 | 4 | 10 | 6 |
| April. | 0 | 2½ | 3 | 2 | 0 | 4 | 9 | 9½ |
| May. | 2½ | 3½ | 2 | 3½ | ½ | 12½ | 3½ | 3 |
| June. | 1 | 5 | 3 | 6½ | ½ | 3 | 6½ | 4½ |
| July. | 0 | 4 | 3 | 0 | 4 | 2½ | 16½ | 1 |
| August. | 2 | 7 | 2 | 1 | 1½ | 3½ | 7½ | 6½ |
| September. | 1½ | 3½ | 1 | 3½ | 2½ | 3½ | 6½ | 8 |
| October. | 3½ | 3 | 0 | 2 | 2½ | 5½ | 13½ | 1 |
| November. | 1 | 1½ | 1 | 2½ | 1½ | 10 | 9 | 3½ |
| December. | ½ | 3 | 1 | 1 | 2 | 8 | 11 | 4½ |
| | 16 | 49 | 20½ | 28 | 19½ | 68½ | 106½ | 57 |

Mean temperature of the year, - - - 51°.308
Maximum height of thermometer, June 13th, - 95°.2
Minimum do. do. Feb. 8, - —17°.0
Range during the year, - - - - - - 112°.2
Warmest day in the year, July 28, mean temp., 82°.1
Coldest day in the year, Feb. 7, mean temp., —5°.2
Mean height of barometer for year, (inches) - 29.3528
Maximum height of barometer, March 4th, - 29.89
Minimum height of do. Nov. 12th, - 28.70
Perpendicular depth of rain and melted snow, (inch.) 52.15
Dryest month in the year, March.
Wettest do. do. June.
Clear and fair days, - - - - - 127
Variable do. - - - - 135
Cloudy do. - - - - - 103
For Winds see the Table.
The Ohio River was not frozen over at this city during the present year.

The year 1835 was remarkable for intensely cold weather, of which there were two periods. The first occurred near the beginning of January, and was felt principally in the North-eastern and Middle states: the second occurred in the early part of February, and was confined chiefly to the Southern and Western states. On the 4th of January at Bangor in Maine, the mercury was frozen; and on the 8th of February the temperature at Lexington, Ky., was 20 degrees below zero. The harvest in the western states was unusually late. According to the statements of some of the oldest inhabitants, so backward a harvest had not been known since the settlement of this country.

JOSEPH RAY, M. D.

2. "*Meteorological Notices in Indiana.* By D. Dale Owen." Silliman's Journal, for January, 1835.

Editorial Note.

In the last number of the American Journal of Science, (a work which should be in the hands of every man of learning and every man of wealth in the United States,) there is a short meteorological paper, most of which we propose to extract into our *Western* quarterly, which *should* be made a repository of all the observations, which the different Meteorologists of the Valley of the Mississippi may record. Mr. Owen's residence is in New Harmony, on the banks of the Wabash, at an elevation above the level of the sea, not quite so great as that of Professor Ray; in lat. 18°. 11', long. 87°. 55':—1° 5' South, and 3° 28' West of Cincinnati. His table embraces observations from the 1st of February, 1828, to the same day of 1829. Three observations were made daily—viz. in the morning soon after sunrise, about one o'clock, and at sunset. It is to be regretted, that while these observations (as far as the thermometer is concerned) may give us very nearly the mean temperature of the day, they do not indicate the extremes, as the greatest depression is, in general, *before* sunrise, and the greatest elevation between two and three o'clock.

"BAROMETER.

| Months. | Mean. | Maximum. | Minimum. | Range. |
|---|---|---|---|---|
| February, | 29.574 | 30.075 | 29.050 | 1.025 |
| March, | 29.583 | 29.886 | 29.150 | 0.736 |
| April, | 29.547 | 29.912 | 29.030 | 0.882 |
| May, | 29.547 | 29.830 | 29.360 | 0.470 |
| June, | 29.538 | 29.660 | 29.290 | 0.370 |
| July, | 29.604 | 29.780 | 29.450 | 0.330 |
| August, | 29.638 | 29.800 | 29.450 | 0.350 |
| September, | 29.688 | 29.890 | 29.490 | 0.500 |
| October, | 29.697 | 29.950 | 29.400 | 0.550 |
| November, | 29.555 | 29.990 | 29.140 | 0.850 |
| December, | 29.746 | 30.100 | 29.270 | 0.830 |
| January, | 29.611 | 30.150 | 29.200 | 0.850 |

| | |
|---|---|
| Mean for the year, - - - - - - | 29.609 |
| Maximum for the year, - - - - - | 30.150 |
| Minimum for the year, - - - - - | 29.030 |
| Range during the year, - - - - - | 1.120 |

THERMOMETER FOR THE SAME YEAR, VIZ. 1828.

| Months. | Mean. | Maximum. | Minimum. | Range. | Days of rain, &c. |
|---|---|---|---|---|---|
| January, | 40.2 | 64 | 12 | 52 | 9 |
| February, | 41.9 | 68 | 12 | 54 | 8 |
| March, | 51.6 | 82 | 20 | 62 | 5 |
| April, | 55.2 | 86 | 28 | 58 | 7 |
| May, | 68.1 | 91 | 42 | 49 | 9 |
| June, | 78.5 | 96 | 55 | 41 | 8 |
| July, | 77.6 | 92 | 53 | 39 | 4 |
| August, | 76.7 | 90 | 63 | 27 | 6 |
| September, | 62. | 84 | 42 | 42 | 3 |
| October, | 55.3 | 79 | 27 | 52 | 2 |
| November, | 44.5 | 64 | 26 | 38 | 4 |
| December, | 40.4 | 63 | 16 | 47 | 3 |

| | |
|---|---|
| Mean temperature of the year, - - - - | 57.7 |
| The lowest, (January) - - - - - - | 12. |
| The highest, (June) - - - - - - | 96 |
| Range for the year, - - - - - - | 84 |
| Greatest range during a single month, (March) | 58 |
| The least, (August) - - - - - - | 27 |
| The number of days, during which any snow or rain fell, (about 1 in 5⅓) - - - - - | 68 |

The most prevalent wind in January, 1828, was W.; in February, W.; in March, S. W.; in April, N. W.; in May, S. W.; in June, S. W.; in July, S. W.; in August, N. W.; in September, N. W.; in October, N. W.; in November, N. W.; in December, N. W.

Almost invariably, the barometer is the highest here, when the wind blows from the N. W., and lowest when it blows from the S. E. As a general rule it rises when the wind blows from the N. W.—N.—N. E., and falls when it blows from the S. E.—E.—S.

The N. W. wind generally brings clear, dry weather, and the atmosphere is then *highly* charged with electricity (particularly in the months of November, December, and January.) The S. W. wind in summer generally brings the thunder storms. The S. E. wind brings the rains of longest duration. The E. and N. E. winds bring the snow storms.

One of the most remarkable meteorological phenomena in this country, is the wonderful electrical state of the atmosphere, and all non-conducting bodies during the autumn and first winter months, when the wind blows from the north west. I have observed, at such times, the hairs of the horses tails so charged, that whole bunches would adhere with considerable force to the horses flanks, or, at other times, the individual hairs, repelled by their neighbors, would stand several inches apart. Distinct shocks, may then be obtained in the fingers, by rubbing the back of a cat with one hand, and holding the point of its tail firmly with the other. One day, last winter, I observed a silk apron attracted by the table so strongly, and at such a distance from it as to enable the wearer to keep it suspended in the air, at a full right angle. When silk or flannel is removed rapidly from the body in the dark, it will exhibit for the moment, one luminous electric sheet, and a distinct crackling noise may be heard. Besides these facts many very interesting electrical phenomena may be observed.

3. *Extract from a "Report on the Recent Progress and Present State of Meteorology.* By JAMES D. FORBES, Esq.," etc. etc., made to the "British Association for the Advancement of Science," at Oxford, in the year 1832.

It was but lately, that we met with the learned and deeply interesting volume, which gives the origin, and transactions for its two first years, of the society just named. It has not been reprinted in America, although it might be circulated here with great advantage. Among other Reports on sciences connected with medicine, is one on *Organic Chemistry*, which, we expect, will be made the basis of an article, for our next number, by our respected colleague, Prof. Rogers, of Cincinnati College. Mean while, we cannot resist the temptation, to place before our readers, in connection with the tables of Prof. Ray, and Prof. Owen, that part of Prof. Forbes' Report, which relates to temperature, (regretting that our limits do not permit us to insert the whole;) and we venture to indulge the hope, that it may stimulate some of them to renewed observations and inquiries. They reside in a country, the atmospheric laws of which are but vaguely comprehended; and could not, therefore, fail to reap a rich reward of *new and striking* results. EDITOR.

TEMPERATURE.

The thermometer is certainly the most perfect of our meteorological instruments. The range of natural temperatures being confined on the surface of our globe within comparatively narrow limits, namely, 96° centigrade or 172° Fahrenheit in the shade*; the indications of the mercurial thermometer may be considered as absolutely accurate. Notwithstanding, too, the difficulties of procuring tubes of perfect calibre, or making due allowance for its variation, the deviations of thermometers made by different makers may, when particular care is taken in their construction, be confined

*Arago, *Annuaire du Bureau des Longitudes*, 1825, p. 186.

within very narrow limits. This I have recently had the means of particularly observing in some comparisons of standard thermometers belonging to the Royal Society of Edinburg, by Professor Christison and myself*. The relation of the mercurial to the air thermometer has been investigated since Gay-Lussac's and Dalton's experiments, by MM. Dulong and Petit, who extended the examination to a great range of temperature. More recently M. Auguste has taken up the subject, and given a formula of comparison†. M. Parrot has re-investigated the subject of the fixed points of thermometers‡. He finds that the purity of the water employed has a sensible influence on its point of congelation; and has observed one tenth of a degree of Reaumur between that of the water of the river Neva and distilled water. He has likewise determined that the maximum heat of water in a state of ebulition occurs at and below seventeen lines under the surface.

Nothing has lately been done in the way of materially improving self-registering thermometers: Rutherford's are still the best. Magnus has proposed one acting by the expulsion of mercury from the end of the tube||,—a proposal made, in the middle of the last century, by Lord Charles Cavendish, and since that revived by Mr. Blackadder§. I have elsewhere taken occasion to point out the principal defects to which it is liable¶. In connection with the thermometer we must mention an elegant method devised by M. Bessel of finding the correction to be applied to any thermometer on account of inequalities in the bore; which consists in breaking off columns of various lengths of mercury in the tube, causing them successively to traverse the length of the scale; and by noting the spaces occupied by each at the different points, an equation for the scale of the particular instrument may be formed**.

A very interesting discovery has recently been made with regard to the early history of the thermometer, by Signor Libri of Florence††. In 1829 were found at Florence a large number of the original alcoholic thermometers made under the direction of the Academia del Cimento, which enabled Sig. Libri to restore the true scale of these early instruments so as to afford a direct comparison with those of modern times. The scale was divided into 50 degrees. The zero corresponded to —15° of Reaumur, the 50th degree with 44° R; and it stood at 13½° in melting ice. The latter fact is interesting because it shows that no sensible change had taken place in the freezing point of those instruments during the lapse of nearly two centuries; for it is recorded that in melting ice the liquid marked 13½°. It is well known that, from whatever cause, many old thermometers indicate a temperature somewhat above 32° Fahr. when plunged in melting ice. In some cases however the change has not

---

* Captain Sabine (*Account of experiments with the Pendulum, &c.* 4to.) found the difference of above a degree in two standard thermometers by the same maker. Such an error however cannot be considered unavoidable.

† Poggendorff's *Annalen*, 1828.

‡ This he has published in a Latin pamphlet, 4to: Petropoli, 1828.

|| Poggendorff's *Annalen*, xxii. 146. § *Edinburgh Transactions*, vol. x.

¶ *Edinburgh Journal of Science*, ix. 300.

** Poggendorff's *Annalen*, 1826. Also *Bulletin des Sciences Mathematiques*, viii. 42., and *Philosophical Magazine*, vol. lxziii. †† *Annales de Chimie*, xlv. 354.

been noticed, and this is one of the most remarkable examples. Some years ago the question excited considerable discussion; but as nothing has been added to our knowledge for some time, I shall merely refer in a note to the papers which have treated of it*. By an accident almost as fortunate as the recovery of the thermometers, some registers nearly complete for sixteen years and kept by Raineri, a pupil of Galileo, have come to light; and by the discussion of them, with a knowledge of the true scale, Signor Libri has been able to show that no sensible change has taken place upon the climate of Florence between that period and the present, which had been suspected.

The metallic thermometers of M. Breguet have not been so much used as might have been expected; they are however rather adapted to delicate experiments on heat than to Meteorology in general. The same remark applies to the beautiful thermo-multiplier recently invented by Signor Nobili†, and which he has applied to the investigation of some of the most delicate phænomena of radiant heat‡.

In the use of the thermometer, the sources of error from terrestrial and solar radiation have not been sufficiently attended to. Some hints on the subject may be found in the article THERMOMETER in the *Edinburgh Encyclopædia*. The mode of exposure of the thermometer of the Observatory at Paris is described by Pouillet in his *Elemens de Physique*.

These various sources of error go far to diminish our confidence in the nice accuracy of thermometric results, where they have not been the subject of particular attention. It is surprising at what a distance a sensible portion of heat is conveyed from soil and walls, or even from grass, illuminated by the sun; the *maxima* of temperature are thus generally too great, and from the near contact in which thermometers are generally placed with large difficultly conducting masses, such as walls, the temperature during the night is kept up, and the *minima* are thus also too high.

This however has been by no means the greatest difficulty in determining the mean temperature of a given spot. Since it is difficult to have the thermometer observed oftener than twice or thrice in a day, it becomes an object of great importance to determine those hours the mean temperatures of which will give that of the whole twenty-four. This however involves an accurate determination of the curve of diurnal temperature; and as this varies with the seasons, its connexion with the curve of annual mean temperature must also be assigned. It is obvious that for these objects a very extended scale of observations is requisite, but that when once attained, the results will be subject to the same general law throughout a considerable extent of country. The mean of the maximum and minimum temperature measured by a register thermometer is one of the best approximations; it is however by no means absolutely accurate. The multiplication of hourly observations has only in one or two instances been resorted to for filling up this blank; but it is to be

---

* Flaugergues, *Bibliotheque Universelle*, xx. 117, xxi. 222, De la Rive and Marcet, *Ibid.* Avr. 1823. Bellani, *Giornale di Fisica*, v. Arago, *Annales de Chimie*, xxxii. Moll, *Edinburgh Philosophical Journal*, ix. 196.

† *Bibliotheque Universelle*, N. S. ii. 225.

‡ *Annales de Chimie*, 1831.

hoped that the facilities which military stations offer for fulfilling this most important object will not be neglected.

The only thermometrical register of great extent which has been undertaken, except that of Toaldo and Chiminello at Padua, and of Neuber at Apenrade, was executed by the military at Leith fort, under the superintendance of the Royal Society of Edinburgh; and an account of the results of complete hourly observations during the years 1824 and 1825 has been published by Dr. Brewster*. The results I consider most important to science; and they afford a proof, which I hope will not be overlooked, that meteorological observations have only to be conducted upon a right scale in order to afford results to a degree of precision scarcely exceeded by any of the physical sciences. The result I particularly allude to is the following. One principal object being to establish the particular hours, the mean of the temperatures of which for the whole year should equal the mean of the whole twenty-four hours, it is obvious that one of these critical times must occur in the morning, the other in the evening. The observations for two years have given the following extraordinary coincidence:

| | Hour of Morning. Mean Temp. | Hour of Evening. Mean Temp. |
|---|---|---|
| 1824 | $9^h$ 13′ | $8^h$ 26′ |
| 1825 | 9 13 | 8 28 |
| Mean | 9 13 | 8 27 |

Such a series of normal observations ought to be made in every extensive country; for the critical hours vary materially with the latitude, and also with the height above the sea. At Paris, for example, the mean temperature occurs *before* 9 o'clock in the morning. At Padua the critical hours were $8^h$ 41′ A. M., and $7^h$ 52′ P. M. But notwithstanding this considerable variation, occasioned by a difference of 11° of latitude, the *interval elapsed* between the morning and evening mean is remarkably constant. At Padua it was $11^h$ 14′; at Leith $11^h$ 12′; and at Apenrade, in Denmark, $11^h$ 11′†. It is to be hoped that the exertions already made by the British Association for the establishment of an hourly register near the equator, and also one in the South of England, will be successful, as the results would be of the highest interest for science.

Some of the other most important consequences deducible from the Leith observations are the following:

The mean hour of minimum temperature for the year is $5^h$ 0′ A. M.; that of maximum temperature, $2^h$ 40′ P. M.

The deviation of any pair of hours of the same name‡ from the mean of the day, is less than 0°·5 Fahr. And of all pairs of hours, 4 A. M. and P. M. are the most accurate.

The reduction of the results of any register made at regular hours, in this climate, to the mean temperature of the day, is readily deduced.

* *Edinburgh Transactions*, vol. x.

† *Schouw Beitrage zur vergleichenden Klimatologie*, 1827.

‡ "Heures homonymes," Humbolt. This author has observed that the same results are deducible from the Padua observations. *Fragments Asiatiques*, ii. 420.

The mean annual temperature of any hour never differs more than 3°·2 from the mean of the day for the whole year.

The mean daily range is a minimum at the winter solstice, snd a maximum in April. The mean daily range in this climate is 6°·065. The result of the two years agree within $\frac{1}{100}$ of a degree, though the mean temperature was considerably different.

By dividing the curve of daily temperature into four parts, by the maximum and minimum points and the points of mean temperature, the intermediate portions of the curve may be accurately represented by parabolic arcs.

On the whole, these most interesting results give us an insight into what may be done, by multiplying observations, towards bringing the science of Temperature under calculation.

An interesting series of comparative observations were undertaken at Christiana under the superintendance of Professor Hansteen, during the warmest and coldest month of the year 1827*. The result is very striking. The daily variation of temperature at Christiana is in February 12°·01 F.; in July, 12°·09. At Leith, in the former it is only 3°·57; in the latter, 9°·68. The annual variation is also immensely greater at Christiana than at Leith:

| | Christiana. | Leith. |
|---|---|---|
| Mean of February - - - | 16°·224 | 40°·621 |
| ———— July - - - - | 61 ·690 | 60 ·361 |
| Difference - - - - | 45 ·466 | 19 ·740 |

These are striking illustrations of the difference of a continental and an insular climate.

The curves of diurnal and annual temperature have been investigated by Professor Hallstrom of Abo, but before the appearance of the above-mentioned observations; he was led to the following equation for reducing the mean of the temperatures at 10 A. M. and P. M. to the daily mean at any part of the year:

$$v = \tfrac{1}{2}\,(xf + xe) - 0{\cdot}33 + 0{\cdot}41 \sin\left[\,(n-1)\,30^\circ + 128^\circ\,8'\,\right]$$

where $v$ is the mean temperature; $\frac{1}{2}(xf + xe)$ the mean of the morning and evening temperatures at 10; and $n$ the number of the month, reckoning from January (= 1). This is intended to apply all over Europe†. M. Poggendorff inquires with some justice, whether it would not be better to avoid all calculation by inclosing the thermometer in a difficultly conducting medium, by which the daily variations of temperature might be diminished.

A mechanical mode of taking the mean of an infinite number of temperatures has been proposed by M. Grassman, by observing the change of rate caused by the influence of temperature upon the uncompensated pendulum of a clock‡. The idea is a good one, but was proposed long ago by Dr. Brewster||.

M. Bouvard's valuable paper upon the meteorological observations at the Observatory of Paris, contains much information, deduced

---

* *Edinburgh Journal of Science*, ix. 309.

† Poggendorff's *Annalen*, 1825. ‡ *Ibid.*

|| *Edinburgh Encyclopædia*, Art. Atmospherical Clock.

from the registers of many years, upon the form of the annual curve of mean temperature at Paris*. He observes that the days of greatest and least temperature in the year are the 14th January and the 15th July, differing only a day from an accurate interval of six months; and each follows the corresponding solstice at an interval of twenty-five days. Baron Humbolt has observed the remarkable symmetry of the curve on either side of its maximum ordinate†. The same author has pointed out the near coincidence of the days of mean temperature observed, even in the too short continuance of registers which we possess‡; these are

Buda, 18th April and 20th October.
Milan, 13th —— and 21st ——
Paris, 22nd —— and 20th ——

These interesting inquiries lead to the general subject of Climatology, which, since the publication of Humbolt's masterly Essay on Isothermal Lines||, has assumed a more satisfactory character than perhaps any other branch of Meteorology. That work has been too long in the hands of every one interested in the particular subject, or in the skillful generalization of groups of facts, to require any notice here; and in touching upon what has been more recently added to our knowledge of the subject, we must confine ourselves within the narrowest limits.

The opinion that the climate of a particular place, or of the globe generally, has materially changed during historic records is improbable; and all the force of that precise and circumstantial evidence which ought to carry weight with it, is against the idea. The eminent naturalist M. Schouw, who has recently published upon several points of interest connected with Meteorology§, has written an Essay, replete with curious matter, upon the supposed change of climate since ancient times, part of which has been translated into English¶, which goes to show that we have no authority for assuming such a change. A similar result has been arrived at by M. Arago, who has collected a number of curious facts relative to great colds which have occurred at Paris, showing, in opposition to an opinion which had been started, that we have no reason to believe the climate to have been deteriorating for some centuries past**. We have already quoted the results obtained by Sig. Libri from the registers of Raineri at Florence.

The old formula of Mayer for expressing the mean temperature of any place in terms of its latitude, which made the temperature of equator 85°, and of the pole 32°, though a respectable generalization

---

* *Memoirs de l' Institut pour* 1824. M. Bouvard has also given en equation for the diurnal curve depending upon the sine of the angle corresponding to the time from noon.

† *Fragments Asiatiques*, ii. 422. ‡ *Ibid.* p. 426.

|| *Memoires d' Arcueil*, tom. iii. p. 462—603.

§ Particularly connected with the geography of plants. See an Essay on the Geographical Distribution of the Vine, *Edin. Phil. Journal;* and an interesting *brochure*, entitled *Specimen Geographiæ Physicæ Comparativæ*, Hauniæ 1828, which contains some interesting camparative views of the climate of the Alps, Pyrenees, and Scandinavian range, the position of the snow line, etc. We have already quoted his work on Oomparative Climatology.

¶ *Edinburgh Journal of Science*, viii. 311.

** *Annuaire du Bureau des Longitudes*, 1825.

for the time at which it was made, was not calculated to stand the experience of another century. Captain Scoresby, I believe, first pointed out its great inaccuracy; and Dr. Brewster, in a paper presented to the Royal Society of Edinburgh, and printed in their Transactions*, showed that the deductions of Humbolt in his Essay on Isothermal Lines would be far better represented by the simple formula

$$p = 81^{\circ}{\cdot}5 \cos L,$$

$p$ being the mean temperature of a place in latitude L. This indeed applies very well to observations made in the meridian of Europe. A more extended view of the subject, and a comparison of the results of Captains Scoresby and Parry, showed, however, that there must exist two poles of maximum cold, one in America, the other in Asia, round which the isothermal lines circulate: and he has lately pointed out, in a letter to Baron Humbolt†, the remarkable analogy of these to the isodymical lines of magnetic intensity, developed by Professor Hansteen. In a private letter dated 27th March, 1830, Dr. Brewster communicated to me his new formula under this form,

$$t = (T + p) \,.\, (\sin^{\frac{1}{2}} d \,.\, \sin^{\frac{1}{2}} d') + p,$$

$t$ being the mean temperature of a place of which the distance from the two cold poles is $d$ and $d'$: T the maximum temperature of the globe, and $p$ the minimum. In the meridian of maximum heat which passes through Europe $d = d'$, and the formula becomes

$$t = (T + p). \sin d + p,$$

which nearly coincides with the formula $T = 81^{\circ}{\cdot}5 \cos L$ given above, and represents observations extremely well. I was much pleased to see a formula which took into consideration the actual *distance*‡ from the cold poles, because it had always struck me that the modifications of the isothermal lines depended upon the accidental figure of the continental masses, it would be better to discard at once the arbitrary coordinates of latitude and longitude, the essential connexion of climate with the latter being nothing, and with the former modified by an infinity of perturbing causes. This seems, in the arbitrary formula just quoted, to be well effected by making the mean temperature a function of the *distance* from the two imaginary poles of greatest cold. Perhaps the modifying circumstances produced by the physical geography of continents are too complex ever to enter expressly into a formuly which should exhibit the relation of the temperature, as an effect, to its really active causes.

Mr. Atkinson has published, in the *Memoirs of the Astronomical Society*||, an examination of the results of Humbolt's researches, with a view to obtain an accurate expression for the law of climate. He has however considered it merely as a function of the latitude, which can never represent universally the phenomena. His equation

$$t = 97{\cdot}08 \cos^{\frac{3}{2}} l - 10^{\circ}{\cdot}53$$

(where $t$ = mean temperature, $l$ = latitude), it will be observed gives

* vol. ix. p. 201. † Poggendorff's *Annalen*, 1831: i. 323.

‡ By employing two distinct formulæ for the two cold poles, Dr. Brewster had before introduced the angle of simple *distance*. See *Edin. Trans.* ix.

|| vol. ii.

the temperature of the equator = 86°·55, and of the pole — 10°·53. The former is decidedly too great, and has been opposed by Baron Humboldt* himself and Dr. Brewster†. It is probable that the mean temperature of the equator does not exceed 81°·5 or 82°.

The temperature of the arctic regions has received the greatest elucidation by the recent labors of Scoresby, Parry, and Franklin. The two last enterprizing travelers have established the existence of a degree of cold quite unsuspected in the northern part of America. From admirably conducted observations, embracing a large portion of the year, the following mean temperatures have been established:

| | Lat. | Mean Temp. | Observer. |
|---|---|---|---|
| Melville Island | 74¾° | — 1½° | Parry. |
| Port Bowen | 73¼ | + 4 | ditto. |
| Igloolik | 69⅓ | + 7 | ditto. |
| Winter Island | 66¼ | + 9½ | ditto. |
| Fort Enterprize | 64½ | + 15½ | Franklin. |

To Captain Beechey also we owe some interesting meteorological results‡.

M. Arago had concluded|| from the results of Scoresby, Parry, and Franklin, that the mean temperature of the pole is — 25° cent., = — 13° F. This however is upon the idea that the cold is at a maximum at the pole, which is not probable; it cannot however be much short of that intense degree. The objection to such a result on account of the supposed increase of ice, which would constantly take place if the temperature were below the freezing point of sea water, I have lately endeavored to combat, and to show that observation presents no opposition to theory§.

A gradual accumulation or facts all over the globe is paving the way for a very accurate knowledge of the mean temperature of its surface; and in a few years more our mass of observations will probably be doubled. Great Britain has done most by her arctic expeditions; and it is earnestly to be desired that with the means she posseses of extending this branch of science likewise in equatorial regions, in the vast continent of India, this great and interesting object will yet meet with some attention, although hitherto totally neglected. In France an excellent register, one of the standard ones in Europe, is kept up, by the assiduity of M. Bouvard, at the Observatory; and several valuable series of observations have been produced by French expeditions in tropical regions. In Switzerland, Meteorology flourishes more than in almost any country in Europe; and though its small extent gives little room for contributions to general climatology, other problems of the greatest consequence have been successfully investigated, as we shall immediately have occasion to notice. From Russia much is to be hoped, for, in the prosecution of the science of Mean Temperature, which we believe is even now obtaining daily accessions of facts from observations in the remote

* *Annales de Chimie et de Physique.* Septembre 1826.
† *Edinburgh Journal of Science*, vi. 117.
‡ Beechey's *Voyage to the Pacific, and Behring's Straits*, 4to edit. vol. ii.
|| *Annuaire*, 1825, p. 186.
§ *Edinburgh Journal of Science*, N. S. v. 17; and *Bibliotheque Universelle*, 1831.

regions of Siberia*; the zeal which has established magnetic observatories in various parts of that vast country†, will not, it is to be expected, neglect the union of some of the most interesting meteorological observations with that of phænomena to which they are so intimately allied. But we wish particularly to allude to the exertions making in the United States of North America to elucidate the mean temperature of that important part of the globe,—one of the most interesting points, indeed, which can at present be examined with a view to rectify our knowledge of the course of the isothermal lines, which, except at the equator, are hardly at all known in the new continent. A great number of Academies scattered over this widely extended country, make annual reports of observations on the mean temperature, fall of rain, and natural phænomena, to the Legislature of New York, and the military stations have afforded extensive series of valuable results‡.

Baron Humboldt has recently published an interesting Essay on the Causes of the Inflexions of the Isothermal Lines||. Without containing much of novelty, this little work gives some general and philosophical views upon climatology, pointing out the nature of the hourly and daily variations of temperature, the variable absorbent and emissive powers with regard to heat of the materials of which the visible surface of the earth is composed; and the changes which cultivation and other circumstances may gradually effect in the elements of climate, especially with regard to the isotheral§ and isocheimal¶ lines, even when the mean annual temperature suffers little variation. The author next considers, at some length, the particular influence of the configuration of soil in modifying the climate, contrasts the continental arrangements of the two hemispheres, and the characters of a terrestrial and marine climate at the equator. He points out in a clear manner the influence of forests upon temperature, from the shadow they produce, from the coolness created by the evaporation of their surfaces, and from the extended radiating surface which they present. Such are the principally interesting points to which this Essay alludes. In the same volume, Baron Humboldt gives some views upon the climate of Asia, which he has collected during his late journey.

The subject of the decrease of heat as we ascend above the surface of the earth, has excited, and especially in Britain, much less attention than it deserves. We hardly know a finer problem for complete solution in Meteorology. Little has been done towards it during the last few years, but we cannot pass it over quite without notice. The principle upon which this diminution of temperature takes place in the higher regions of the atmosphere, is now universally allowed

* See Humboldt's *Address to the Petersburg Academy of Sciences*, 28th Nov,, 1829.

† Humbolt, Kupffer.

‡ *Edinburgh Journal of Science*, viii. 303. x. 267, etc.—In alluding to the exertions of different Governments for the advancement of meteorological science, I am happy to be able to add that of Prussia. I have had the good fortune to meet this summer (1832,) in the Alps, M. Kamtz, a zealous member of the University of Halle, who had been sent on a scientific mission by the Prussian Government to establish some most curious facts in Meteorology.—J. D. F., Dec. 1332.

|| In his *Fragments Asiatiques*, tom. ii. p. 398.

§ Equal summer temperature.

¶ Equal winter temperature.

to be the increased capacity for caloric of air when it is rarefied. The first question for solution is, therefore, the specific heat of air under different degrees of condensation,—a point of by no means easy investigation, and which has engaged the attention of some of the first philosophers of the day, as we have already noticed. It has been investigated experimentally by Dalton, and Leslie, De la Rive, and Marcet; and theoretically by Laplace, Ivory, Poisson, and Avogadro. The object is the more important, as it is intimately connected with the amount of astronomical refractions. We do not consider, however, that the total effect can be expressed simply by the law of specific heats varying with height; and it appears that the experimental data which have been sought by observations on the atmosphere, have not generally been conducted on principles which can lead to conclusive results. I do not allude merely to the use of *insulated* observations of temperature at great elevations, which in my opinion can lead to no general result*, because of the innumerable accidental causes always at work, which can only have their influence multiplied by a long series of observations,—but to the fact, that observations of temperatures at great heights, whether on insulated points or in balloons, have been directly compared with those close to the massive heating surface of the earth, the radiation of heat from which probably exerts far more influence in producing a decrease of temperature in the very lowest strata of the atmosphere, than the general law which is sought for, and which is usually alone considered. The true law of decrease of temperature, such as it would be *if the earth was removed*, must be sought for probably by successive stages of balloon observation†, *commencing* at a considerable height above the surface; or else we must find the means of estimating accurately the calorific influence of the earth by radiation and conduction. There cannot be a question that in the lowest strata the diminution of temperature appears much greater than at higher elevations, if the observations be not made on a naturally inclined surface of soil, but at two stations, one nearly vertically above the other‡.

In order that the terrestrial influence may be as much equalized as possible, observations on the mean temperature of table lands at considerable elevations, (but not covered with perpetual snow, which introduces a new element,) are perhaps the most satisfactory. And hence the reason that Humboldt's equatorial observations are by far the best that we possess||. Humboldt's general result gives 121 toises of ascent for a diminution of 1° R. The admirable comparative observations at Geneva and St. Bernard, give a surprisingly near approach to this, the difference of mean temperature of the stations being 8°·64 R. for 1069 toises: this gives 123½ for 1° R. or 352 feet for 1° Fahr. This probably is the most correct mean result which we can

---

* Such as the collection of insulated observations in Ramond *Sur la Formule Barometrique de la Mecanique Celeste*, p. 189:

† A few observations with balloons have been published by Lord Minto (*Edinburgh Journal of Science;*) it will be necessary however to carry them on upon a much more extended scale, before any general conclusions can be attained.

‡ See some observations, remarkably confirmatory of this view, by Sir Thomas Brisbane, (made in New South Wales,) in the *Edinburgh Journal of Science.*

|| *Observations Astronomiques*, 4to, i. 126.

adopt. The influence of the seasons is very considerable, as the following results of M. Guerin at Ventoux near Avignon, lately published, prove*.

Summer, 80 toises for 1° R., or 156 metres for 1° cent.

Winter, 100 ————— or 195 —————

Some good observations made in summer upon the Rigi by M. Eschmann of Zurich, give 97 toises of ascent for 1° R.† I have succeeded in establishing, in latitude 56°, a system of observation on this intersting point, from which I hope in due time to be able to obtain the most important results.

Several formulæ have been proposed for a general expression of the law of gradation. Lagrange, and many after him, considered the decrement of heat to be simply proportional to the height, and the best observations may be tolerably represented by

$$t = n\,\mathrm{H}.$$

Where $t$, the decrement of temperature, is expressed in degrees of Fahrenheit, and H in English feet, the coefficient $n$ will be a fraction nearly equal to $\frac{1}{270}$, or between that and $\frac{1}{300}$, for *small* heights where the decrement proceeds most rapidly: the observations of Humboldt and those at the Grand St. Bernard would, we have just seen, make $n$ about $\frac{1}{350}$. Euler considered the progression an harmonical one. Professor Leslie, from experiments upon the heat absorbed by air in rarefaction, proposed theoretically the formula $25\left(\frac{1}{s} - s\right)$ for the diminution of temperature on the centigrade scale; where $s$ represents the density of the air at the upper station. This formula was first proposed without demonstration‡; afterwards the nature of the experiments upon which it rested was explained||: from its principle, this formula only takes cognizance of the influence of the change of specific heat in the atmosphere, without any reference to the effect of the mass of the earth. Professor Leslie's formula has given rise to several discussions, to which it will only be necessary to refer in a note§.

Mr. Atkinson, in discussing the subject of Astronomical Refractions¶, has examined with great care all the actual observations to which he had access, and from them he has deduced the following formula:

$$h = [251{\cdot}3 + \tfrac{3}{2}(n - 1)]\,n,$$

where $h$ is the height of the station in English feet, $n$ the depression of Fahrenheit's thermometer.

An ingenious attempt was made by M. Mathieu** to deduce the law of decrease of temperature in the polar regions, by analysing

* *Annales de Chimie*, xliii. 428. † *Bibliotheque Universelle*, 1827.

‡ Leslie's *Elements of Geometry*.

|| *Encyclopediæ Britannica*, Supplement, Article Climate. See also Thomson *On Heat* p. 122.

§ By Mr. Ivory, *Philosophical Magazine*, 1821; by an anonymous writer, *Edinburgh Journal of Seience*, vol. v.; and a paper by myself relative to the last-mentioned one, *Ib.* N. S. vol. v.

¶ *Memoirs of the Astronomical Society*, vol. ii.

** Humboldt, *Observations Astronomiques*, i. 155.

two observations by M. Swanberg upon the amount of refraction, by means of the formula of the *Mecanique Celeste.* The result at which he arrived was 243 metres of elevation for a decrease of 1° cent. This however is at variance with the only direct observation we have on the subject. Captain Parry found in latitude 69° 21′, by means of a kite, that the thermometer indicated no diminution of temperature at a height of 400 feet; it stood at —24°. Much however cannot be inferred from a single observation.

Some observations have recently been made by M. Kupffer in the range of the Caucasus, by means of the temperature of springs, but too few to admit of any satisfactory conclusions*.

We must now touch upon a point of the greatest importance, and which daily increases in interest,—I mean the proper temperature of the globe itself. We have already pointed at the fine views of Baron Fourier relating to this subject; and from the experimental confirmation which they receive every year, there is little doubt that they will soon be established on an immovable basis. He considers the globe as a mass in the process of cooling from an intense temperature. He has proved that the heat may be very intense at a short distance from the surface, and yet from the extremely bad conducting power of the crust, that it may exert no sensible influence on the climate: he actually computes it as not amounting to one thirtieth of a centigrade degree. Towards the centre the heat may be of the most extreme intensity, and the phænomena of earthquakes and volcanoes may be imputed to its influence. The process of cooling, though at first of course comparatively rapid, may now be considered to have reached an asymptotic condition. It is well known that the influence of the seasons, or the total difference of the effect of solar radiation in summer and winter, affects the temperature of the soil to a comparatively minute depth. Experiments with thermometers, sunk to different depths, have been made at Zurich by M. Ott, near Edinburgh by Mr. Ferguson, and at Strasbourg by M. Herrenschneider†. The influence of the solar rays decreases rapidly; and it is probable, from experiments made at Paris‡, that at about 30 metres, or 100 feet, it is almost extinct. The position where this takes place is called by Fourier the "couche invariable," or *invariable stratum;* all variations above this plane are imputed to the influence of radiation, all below to the native or primæval heat of the globe.

A successive influx and efflux of heat is constantly going on in the strata above that of invariability. The heat of the solar rays is constantly acting on it during the day, and with an intensity depending upon the absorbent power of the surface, and the latitude. M. Pouillet, from some ingenious experiments, concludes that the solar rays which reach the surface of our globe in the course of a year, have sufficient intensity to melt a complete stratum of ice over its whole extent of 14 metres in thickness‖. The amount of solar radiation in various parts of the globe, presents almost an open field for investigation. Mr. Daniell, in his *Meteorological Essays*, started the

* *Voyage au Mont Elbrontz*, 4to, Petersburg.

† See a *resume* of these experiments by M. Pouillet; *Elemens de Physique*, ii. 642.

‡ The excellent observations regularly made in the caves under the Observatory are regularly published in the *Annales de Chimie et de Physique.*

‖ *Elemens de Physique*, ii. 704.

apparently paradoxical opinion that the force of solar radiation increases from the equator to the poles; and though his reasonings have been opposed by an eminent French philosopher*, and by meteorologists at home†, we think he has at least had the merit of pointing out the fact, that the force of radiation is much less in the equatorial and much greater in the polar regions than might have been anticipated. Dr. Richardson has made some very interesting though not quite decisive experiments on this subject in the late Northern expedition‡. I am indebted to Mr. (now Sir John) Herschel, for the remark, that observations on solar radiation seem generally to have been made upon an erroneous principle; the true indication of the force of the solar rays, not being the statical effect upon the thermometer, but their momentary intensity mesured by the velocity with which they communicate heat to an absorbent body. We may confidently point out the subject of radiation as one which will reward the researches of the Meteorologist.

When the immediate calorific cause no longer acts, the surface of the globe of course begins to radiate the superfluous heat which it had received, and exactly in proportion to the facility with which it received it, the absorbent and emissive powers of surfaces being equal. Hence a nocturnal radiation of heat takes place from the soil, occasioning that cold which, according to the laws known to regulate this process, is materially affected by the purity of the sky; for it is perfectly certain that clouds or any interposed body, or even the finest films, have a sensible influence in intercepting the rays of invisible heat. To the coolness thus produced in bodies exposed at night to a clear sky, the phænomena of dew have been accurately attributed by Dr. Wells in his Essay on that subject,—a work undoubtedly both philosophical and important, but which it appears to me has received fully its due share of commendation.

Let us now see what experimental proofs we have of native heat below the invariable stratum. It is nearly a century since it was first suspected that the temperature of the earth increased as we descend. The proof however has been reserved for our own day, by the multiplication of observations which might annihilate every plausible objection,—for many such there undoubtedly are. Nor can we assert, that before the late researches of M. Cordier‖ truth has decisively been made to appear. The general consequences which have resulted from his inquiries, and which are substantiated by an abundant collection of facts observed in Cornwall, Saxony, Brittany, Switzerland, America, and other points, are as follow:—That the temperature of any stratum below that of invariability is absolutely the same all the year round§;—That in all strata so situated the temperature increases as we descend, *without any exception;*—That though the results which have been obtained are far from giving the same law of increase for different countries, which from the imperfection of the observations it was impossible to expect, yet the

---

* *Annales de Chimie*, Aug. 1824. † *Edinburgh Philosophical Journal*, xiv.

‡ Franklin's *Second Journey*, 4to edit.

‖ *Annales du Museum d'Historie Naturelle*, 1827. See also *Bulletin des Sciences Mathematiques*, ix. 111; *Edin. New Phil. Journal*, vol. v. & vi.; and De la Beche's *Manual of Geology* (Introduction.)

§ See proofs of this in Saxony, *Annales de Chimie*, xiii. 211.

general progression may be stated at from 25 to 30 metres of descent for an increase of one degree centigrade, or from about 37 to 44 feet for one degree Fahr.* M. Cordier has elaborately and successfully refuted the idea, that these effects could be produced by the lamps of the miners, though he has shown the nature and amount of the influence they actually exert. A more refined objection, imputing the heat to the condensation of atmospheric air descending into the mines, has been satisfactorily answered by Mr. Fox†, whose scientific observations on the mines in Cornwall, and especially on their temperature, and the electro-magnetic properties of their metaliferous veins, promise so much towards the advancement of science. M. Magnus, whose register thermometer we have already alluded to, and who applied it to the present object, has recently published some good observations on a boring near Berlin‡. The temperature of the earth, measured by that of deep-seated and copious springs, has conducted to a similar result; for their variation of temperture as connected with latitude is confined within smaller limits than the mean temperature of the air, indicating a *proper temperature* of the earth, which at the mean depth of springs diminishes that of those near the equator, and increases it near the poles; the mean point, or where the ordinary temperature of the earth and that of the air are the same, being about latitude 56°. Von Buch pointed out some years ago this interesting fact‖, which was deduced from his own observations and those of others, especially of Wahlenberg in Sweden§. The question has lately been treated in its greatest generality by M. Kupffer, who has established to a considerable extent the course of what he calls the isogeothermal lines, and has given formulæ for their computation as well for longitude as latitude; for, like Humboldt's isothermal lines, he finds that they do not regularly follow the parallels of latitude, but are subject to anomalous inflections. The form of the expression given by M. Kupffer is

$$a - b \sin^2 l = t,$$

$a$ and $b$ being constants which vary with the meridian of the place, and which he has computed for a range extending from 85° W. to 60° E. of Paris¶. Near the equator, the ground at 25 metres depth appears to have a temperature 2° R. *below* the mean temperature of the air, whilst in Lapland it is as much *above* it. I do not think that the connection of this remarkable fact with the proper temperature of the globe has been pointed out with sufficient distinctness, but this is not the place to insist upon it more particularly.

It is obvious that the temperature of the ocean, which covers so large a portion of the surface of our globe, must have a great

* M. Kupffer has lately deduced 36°·81 English feet for 1° F. Poggendorff's *Annalen*, xv. *Edinburgh Journal of Science*, April 1832. The recent experiments of M. Gherard in Prussia, communicated by Baron Humboldt to the Academy of Sciences, give 180 feet of descent for 1° R. *Bulletin de la Societe Philomathique.* Mars—Avr. 1832.

† *Philosophical Magazine* 1830. Both to Mr. Fox and Mr. Henwood we are indebted for some excellent original experiments. *Edinburgh Journal of Science*, vol. x.

‡ Poggendorff's *Annalen*, xxii. 146.

‖ See *Edinburgh New Philosophical Journal*, vi. 166.

§ Wahlenberg's observations were orginally published in Gilbert's *Annalen der Physik.*

¶ *Voyage au Mont Elbrontz.*

influence in the modification of its climate. This subject has therefore occupied particular attention. The most active observers have been Humboldt, Scoresby, Parry, Ross, Sabine, Hall, Davy, and Duperrey. The variations of temperature of the sea being comparatively small, the climate is subject to much smaller fluctuations than on continents. As might be expected, the maximum temperature of the air is greater than that of the surface water; the mean temperature however, it appears from the observations of Duperrey, is somewhat less. The temperature, as it varies with depth, is of course amenable to the laws of the maximum density of water; though with respect to *salt* water, this phænomenon still requires elucidation*. Within the tropics, the temperature constantly diminishes as we descend. Towards latitude 70° this decrease vanishes, and an opposite series of phænomena take place; the temperature increasing as we descend. As these points, though interesting in a high degree, are not so intimately connected with Meteorology as those we have been discussing, and those which yet remain, we shall merely give references to those works where general views have lately been given on the temperature of the sea:—Arago, *Annuaire pour* 1825; Humboldt, *Relation Historique*, iii. 514—530, and *Fragments Asiatiques*, ii. 556; Pouillet, *Elemens de Physique*, ii. 684; Recent Observations by M. Lens, who accompanied Kotzebue; Poggendorff's *Annalen*, 1830; and the *Voyages* of Beechey and Duperrey.

* From Erman's experiments it would appear that sea water has no point of greatest density above its freezing point. *Annales de Chimie*, xxxviii. 287.

